# Design and Experimental Validation of a Round Inductosyn-Based Angular Measurement System

**DOI:** 10.3390/mi17010005

**Published:** 2025-12-20

**Authors:** Jian Wang, Jianyuan Wang, Jinbao Chen, Chukang Zhong, Yuankui Shao

**Affiliations:** 1 College of Astronautics, Nanjing University of Aeronautics and Astronautics, Nanjing 211106, China; bx2315004@nuaa.edu.cn (J.W.);; 2 National Key Laboratory of Aerospace Mechanism, Nanjing University of Aeronautics and Astronautics, Nanjing 211106, China

**Keywords:** round inductosyn, angular measurement system, resolver-to-digital converter, coarse–fine data fusion, linear interpolation error compensation

## Abstract

This paper presents the design, implementation, and experimental validation of a high-precision angular measurement system based on a round inductosyn. Dedicated hardware circuits, including excitation, signal conditioning, and resolver-to-digital conversion modules, together with software algorithms for coarse–fine data fusion and linear interpolation-based error compensation, are developed to achieve accurate and stable angular measurement. Experimental results obtained from repeated measurements over a full rotation demonstrate reliable system operation and effective suppression of nonlinear errors. After compensation, the residual angular error is limited to within ±3″, while measurement consistency across repeated experiments is significantly improved. The output angle exhibits good continuity and stability, confirming the feasibility and effectiveness of the proposed system for high-precision servo control and aerospace attitude measurement applications.

## 1. Introduction

Precise angular measurement plays a fundamental role in modern servo systems, aerospace attitude control, robotics, and precision instrumentation, where the accuracy, stability, and real-time response of angular feedback directly determine overall system performance and reliability [1,2,3]. Common angular position sensors include optical encoders, resolvers, capacitive sensors, and inductosyns [4,5,6,7]. Among these, optical encoders can provide very high resolution; however, their performance is highly sensitive to environmental factors such as temperature variation, vibration, contamination, and electromagnetic interference, which may lead to accuracy degradation or signal loss under harsh operating conditions [8,9].

Inductosyns, based on the principle of electromagnetic induction, offer contactless operation, strong environmental adaptability, and excellent long-term stability, making them particularly suitable for aerospace and other high-reliability servo applications [10,11,12]. In particular, the round inductosyn employs a circular winding structure to generate two orthogonal sinusoidal induced signals whose amplitudes vary periodically with rotor position. After appropriate signal conditioning and computation, continuous angular measurement with high potential resolution can be achieved. Owing to these advantages, round inductosyns have been widely investigated and applied in precision servo mechanisms, aerospace actuation systems, and harsh-environment sensing scenarios [13,14].

From a system implementation perspective, existing inductosyn- and resolver-based angular measurement systems can be broadly classified into three main categories. The first category relies on purely analog demodulation architectures, in which sine and cosine signals are processed through phase-sensitive detection and analog filtering circuits to extract angular information. Although these systems are conceptually simple, their performance is strongly affected by component tolerances, phase drift, and noise, making it difficult to achieve stable second-level accuracy in practical applications [15,16].

The second category adopts hybrid analog–digital architectures, typically using resolver-to-digital converter (RDC) chips to perform synchronous demodulation and closed-loop tracking. This approach improves noise immunity and simplifies digital processing, and has been widely adopted in industrial drives and precision motion control systems [17,18,19]. However, the achievable accuracy of such implementations still depends heavily on excitation stability, weak-signal conditioning quality, and synchronization consistency between sine and cosine channels.

The third category focuses on digital calibration and error compensation, aiming to mitigate periodic nonlinear errors caused by winding asymmetry, magnetic field nonuniformity, and amplitude–phase imbalance through modeling and algorithmic compensation [20,21,22]. Studies on arctangent-based angle estimation have shown that even small amplitude or phase mismatches between orthogonal signals can introduce dominant harmonic errors, which fundamentally limit achievable accuracy if not properly compensated [23,24].

Despite these advances, several challenges remain in the practical application of round inductosyn-based angular measurement systems. First, the induced sine and cosine signals are typically at the millivolt level, making them highly susceptible to external noise during transmission and amplification, especially in electrically harsh environments [25]. Second, imperfections such as winding asymmetry, magnetic path nonuniformity, and phase deviation between sine and cosine channels introduce periodic nonlinear errors that degrade angular accuracy. Third, dual-channel round inductosyns, which combine coarse and fine windings to achieve both wide measurement range and high resolution, require precise synchronization and robust data fusion strategies to avoid ambiguity and output discontinuities. However, many existing studies address these issues in isolation and lack system-level integration between hardware circuit design and software compensation algorithms, limiting overall measurement performance and practical applicability [26].

To address the above challenges, this study presents a fully integrated round inductosyn-based angular measurement system with a coordinated hardware–software architecture. The proposed system incorporates a dedicated excitation power amplifier, cascaded filtering and amplification circuits for weak induced signals, and RDC-based synchronous demodulation modules. On the software side, real-time RDC driving, dual-channel coarse–fine data fusion, and linear interpolation–based error compensation are implemented to achieve continuous and unambiguous angular output. Through the joint optimization of hardware signal integrity and digital processing, the system enables accurate conversion of analog sine–cosine signals into digital angle values while effectively mitigating periodic nonlinear errors inherent to the inductosyn.

The contribution of this study lies in the system-level integration, coordinated design, and comprehensive experimental validation of a complete round inductosyn angular measurement chain under practical operating conditions. Experimental results demonstrate that, after compensation, the residual angular error is confined within ±3″, and the output angle exhibits excellent continuity and stability, confirming the effectiveness of the proposed system architecture.

The main contributions of this work can be summarized as follows.

1. A fully integrated round inductosyn-based angular measurement system is developed, including a dedicated excitation power amplifier, cascaded filtering and amplification circuits for weak induced signals, and RDC-based synchronous demodulation.
2. A dual-channel coarse–fine data fusion strategy is implemented to combine the wide measurement range of the coarse channel with the high resolution of the fine channel, enabling continuous and unambiguous angular output.
3. A linear interpolation–based error compensation method is applied to mitigate periodic nonlinear errors of the inductosyn, improving angular accuracy and repeatability.
4. Comprehensive experimental validation is conducted on a practical platform, demonstrating an angular measurement accuracy within ±3″ after compensation, as well as stable and continuous output performance.

The remainder of this paper is organized as follows. Section 2 introduces the operating principle of the round inductosyn and the corresponding signal processing mechanism. Section 3 presents the hardware and software design of the proposed measurement system. Section 4 describes the experimental setup and validation results. Finally, Section 5 concludes the paper and discusses future research directions.

## 2. Principle and Computation Method

### 2.1. Operating Principle of the Round Inductosyn

The round inductosyn is a multi-stage position feedback device based on the principle of electromagnetic induction. It accurately determines angular position by utilizing the periodic variation in the induced alternating electromotive force generated by the relative rotation between the stator and rotor.

**Figure 1:**
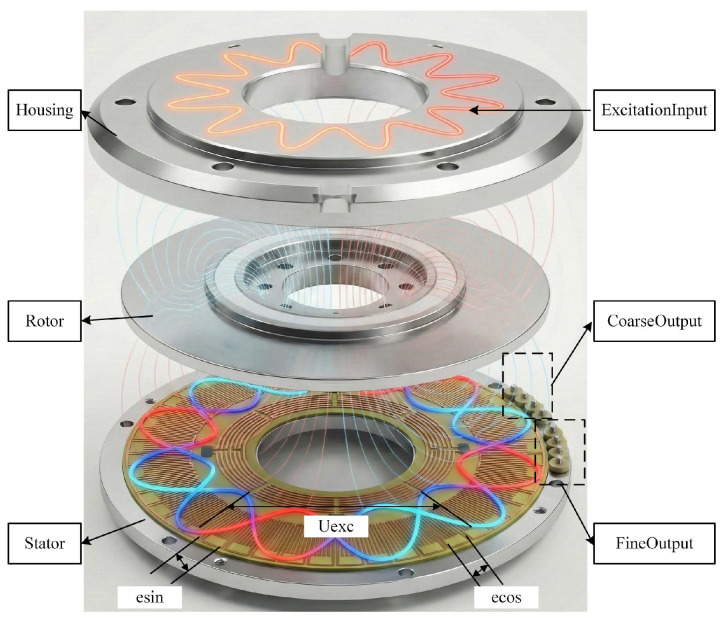
Mechanical structure and operating principle of a round inductosyn with sinusoidal excitation and orthogonal induced signals.

As shown in Figure 1, both the conductors and their spacing within the stator and rotor windings are arranged in a fan-shaped pattern. The rotor winding is continuous and connected in series through conductors distributed along its inner and outer end surfaces. In contrast, the stator winding is divided into multiple segments, forming two orthogonal groups of windings corresponding to the sine and cosine functions. Each segment of the stator winding is internally connected in series via end-face conductors, and the outermost conductor of each segment is led out and interconnected to form the complete sine and cosine windings.

When a sine excitation signal of a specific frequency is applied to one of the windings, an alternating electromotive force is induced in the corresponding winding. Both phase windings are arranged on the same substrate. The continuous winding identifies the current rotation period of the rotor, while the segmented winding provides precise positional information within that period. Together, they form an absolute position measurement system.

**Figure 2:**
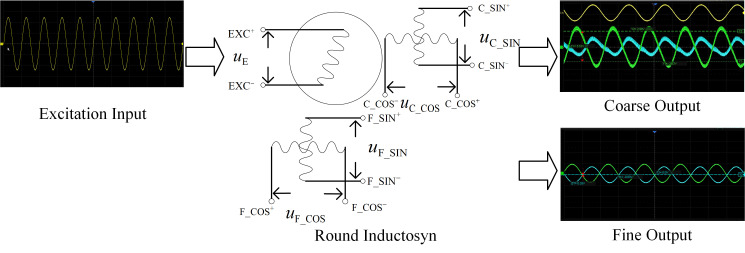
Electrical operating principle and signal generation of the round inductosyn.

As illustrated in the following Figure 2, EXC denotes the input excitation signal, C_SIN and C_COS represent the sine and cosine induced signals of the continuous winding, and F_SIN and F_COS represent those of the segmented winding.

When a sinusoidal excitation signal as expressed in Equation (1) is applied to the terminals of the continuous winding, where *Um* denotes the amplitude of the applied voltage and *ω* represents the angular frequency of the excitation signal [10,11,12,13].

(1) $$u(t)=U_{m}Sin\omega t$$

At this moment, the induced signals can be expressed as shown in Equation (2):

(2) $$\left\{\begin{array}{c} U_{C_{S\mathrm{IN}}}=K_{u}U_{m}\sin(\omega t)\sin(\theta_{C})\\ U_{C_{COS}}=K_{u}U_{m}\sin(\omega t)\cos(\theta_{C})\\ U_{F_{SIN}}=K_{u}U_{m}\sin(\omega t)\sin(\theta_{F})\\ U_{F_{COS}}=K_{u}U_{m}\sin(\omega t)\cos(\theta_{F}) \end{array}\right.$$

where *U_CSIN_* and *U_CCOS_* represent the sine and cosine induced signals of the continuous winding, and *U_FSIN_* and *U_FCOS_* denote those of the segmented winding. *K_u_* is the voltage transfer coefficient, *U_m_* is the amplitude of the excitation voltage, Ω is the angular frequency of the excitation signal, and *θ_C_* and *θ_F_* correspond to the periodic position of the continuous winding and the absolute position of the segmented winding, respectively.

**Figure 3:**
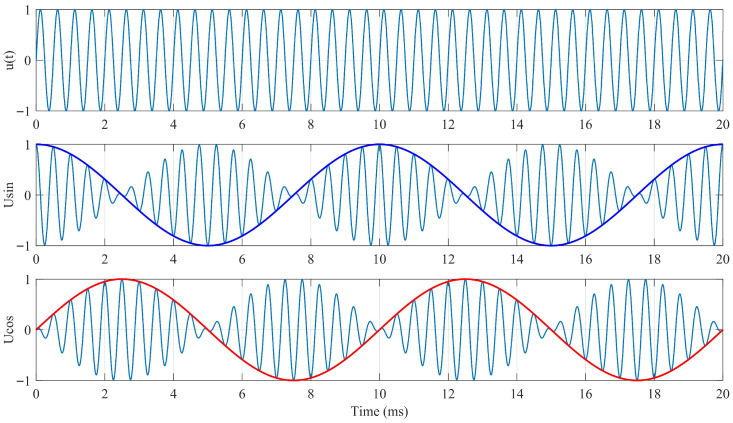
Induced EMF Waveform of the Round Inductosyn.

A sinusoidal excitation signal with an amplitude of 5 V and a frequency of 2 kHz is applied to the continuous winding. The voltage transfer coefficient *Ku* is set to 1, and the rotational speed is configured as 50°/s. The excitation signal and the two induced phase signals are shown in the following Figure 3, where the blue and red envelopes correspond to the position-dependent components *sinθ* and *cosθ*, respectively.

### 2.2. Angle Computation Method

The angle computation methods of the round inductosyn can be categorized according to the characteristics of the induced signals and the excitation mode. Based on whether the angle information is derived from the amplitude or phase of the induced signal, the methods are classified into amplitude-detection and phase-detection types. In addition, depending on the excitation configuration, they can be further divided into continuous-winding excitation and segmented-winding excitation. Consequently, four possible angle computation schemes can be defined.

In the phase-detection method, the excitation signal is phase-shifted by 90° to serve as the reference signal for position calculation. The phase difference between the induced signal and the reference signal corresponds to the measured angular position. However, the accuracy of this approach is highly dependent on the system frequency, and the precise realization of a 90° phase shift is difficult, leading to inherent systematic errors.

As shown in Equation (2), the excitation and induced signals are in phase, but the amplitude of the induced voltage varies with the mechanical angle. The amplitude-detection method estimates the angular position by sampling the signal amplitudes and processing them through specific computational algorithms. Since the continuous-winding excitation requires only a single power-amplified sinusoidal source, this design adopts the amplitude-detection method with continuous-winding excitation for angle signal demodulation.

Given that Equation (1) defines the input excitation signal, a phase-shift operation is applied to obtain a reference signal *φ* with known angular information. By multiplying the induced signals of the coarse and fine channels by *φ* and subsequently taking their difference, the following relationship can be derived.

(3) $$\left\{\begin{array}{l} U_{\mathrm{Ce}}=k_{u}U_{m}\sin(\omega t)\left(\sin\theta_{C}\cos\phi -\cos\theta_{C}\sin\phi \right)=k_{u}U_{m}\sin(\omega t)\sin\left(\theta_{C}-\phi \right)\\ U_{\mathrm{Fe}}=k_{u}U_{m}\sin(\omega t)\left(\sin\theta_{F}\cos\phi -\cos\theta_{F}\sin\phi \right)=k_{u}U_{m}\sin(\omega t)\sin\left(\theta_{F}-\phi \right) \end{array}\right.$$

Here, both the excitation signal *sin(ωt)* and the reference signal *φ * are known. After demodulation, the following expressions can be obtained.

(4) $$\left\{\begin{array}{c} U_{eC}^{\prime }=\sin\left(\theta_{C}-\phi \right)\\ U_{eF}^{\prime }=\sin\left(\theta_{F}-\phi \right) \end{array}\right.$$

**Figure 4:**
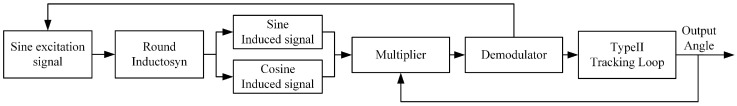
Schematic Diagram of the Type-II Tracking Loop.

At this stage, a Type-II closed-loop tracking system, as illustrated in the following Figure 4, is constructed. The characteristics of the Type-II system ensure zero steady-state error in tracking the input signal while limiting the system bandwidth. Under these conditions, U′*_eC_* and U′*_eF_* approach zero, and the system output becomes equal to the input, representing the true angular value.

## 3. System Design

### 3.1. Hardware Architecture

**Figure 5:**
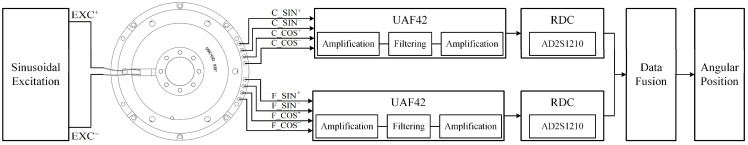
Flowchart of the Round Inductosyn Decoding Process.

The angular measurement method adopted in this study is the tracking feedback approach, and its overall structure is shown in the following Figure 5. Functionally, the system can be divided into four main circuits: the excitation signal power amplifier, the induced signal filtering and amplification circuit, the resolver-to-digital conversion circuit, and the controller-to-host communication circuit. The controller configures the RDC chip to generate a fixed-frequency sinusoidal excitation signal, which is amplified by the power amplifier and applied to the round inductosyn. The excited inductosyn produces millivolt-level sine and cosine signals, which are then processed through two stages of amplification and band-pass filtering before being sent to the RDC demodulation chip. After demodulation, the chip transmits the calculated position value to the controller via the SPI communication protocol. The controller performs data fusion and subsequently transfers the angular position to the host computer through serial communication.

Each RDC chip can decode only one pair of differential sine and cosine signals. Therefore, to realize synchronous decoding of the dual-channel round inductosyn, two RDC chips are connected in parallel and share a common clock signal. One chip outputs the excitation signal, while the excitation output of the other chip remains unconnected. A hardware design with equal-length signal paths is implemented to ensure real-time synchronization, enabling simultaneous decoding of both coarse and fine channels of the round inductosyn and converting them into digital position outputs.

#### 3.1.1. Power Amplifier Design for Excitation Signal

**Figure 6:**
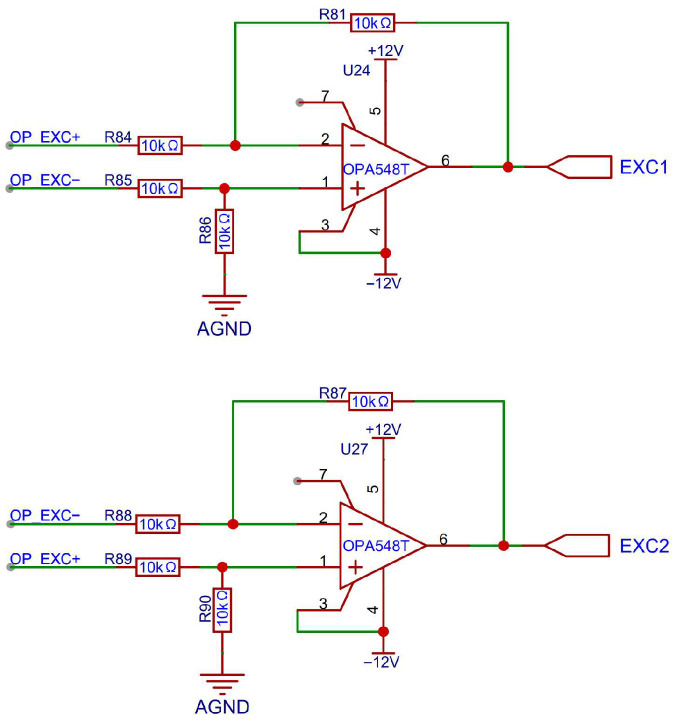
Circuit Diagram of the OPA548T Power Amplifier.

In this design, the excitation signal is a 10 kHz differential sine wave directly generated by the RDC chip. Since the round inductosyn is a resistive component whose resistance is much greater than its reactance, a driving current of at least 1 A is required for proper operation. For differential driving, two high-power operational amplifiers OPA548T (Texas Instruments, Dallas, TX, USA) are employed in a symmetrical configuration, providing an output voltage range of ±10 V and a maximum excitation current of up to 3 A. Because the amplitude of the excitation signal from the RDC chip already meets the required voltage level for the inductosyn, the power amplifier design focuses primarily on current amplification rather than voltage gain. The detailed implementation circuit is shown in Figure 6.

The power amplification circuit consists of a differential amplifier with two input signals of opposite phase. It generates sinusoidal outputs with equal amplitudes and opposite phases. Under a constant output voltage, each amplifier handles only half of the total current, and since no current flows through the ground line, ground potential fluctuations are reduced. This configuration simplifies the circuit design and simultaneously enhances thermal performance.

#### 3.1.2. Induced Signal Filter and Amplifier Design

When excited, the inductosyn generates two-channel sine and cosine signals, producing four outputs in total. Each signal has an amplitude ranging from 3 to 10 mV, whereas the RDC chip requires an input voltage of approximately 3.2 ± 0.6 Vpp. Therefore, the signals must be amplified by about 1000 times, demanding amplifiers with high gain and low noise characteristics. Direct single-stage amplification would excessively amplify system noise and cause waveform distortion. To achieve the desired overall gain while maintaining a high signal-to-noise ratio, the proposed design employs a cascaded structure consisting of a first-stage amplifier, a band-pass filter, and a second-stage amplifier.

**Figure 7:**
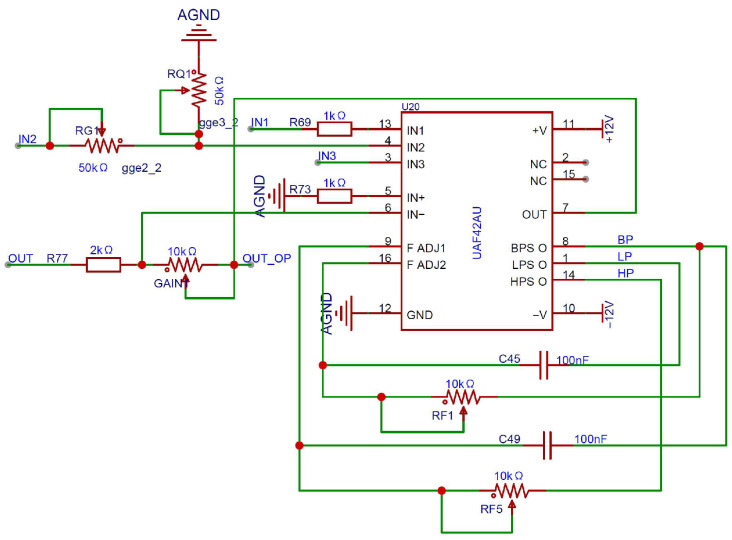
Schematic of the UAF42-Based Signal Processing Circuit.

As shown in Figure 7, the core component of the induced signal processing circuit is the active filter chip UAF42, which is configured in the band-pass filtering mode through jumper settings [25]. Under this configuration, the center frequency is determined as expressed in Equation (5).

(5) $$\omega_{n}^{2}=\frac{R_{2}}{R_{1}R_{F1}R_{F2}C_{1}C_{2}}$$

Here, *R*_1 _= *R*_2_ = 50 kΩ and *C*_1_ = *C*_2_ = 1000 pF are fixed internal components, while *R_F_*_1_ and *R_F_*_2_ are external adjustable resistors. The center frequency can be tuned by varying these resistors. During adjustment, *R_F_*_1_ and *R_F_*_2_ should be kept as close in value as possible to maintain a high Q-factor and stable center frequency, which can reach up to 40 kHz. At this point, the gain of the band-pass filter can be easily adjusted, as expressed in Equation (6).

(6) $$A_{BP}=\frac{R_{4}}{R_{G}}$$

Here, *R*_4_ is an internal fixed resistor with a value of 50 kΩ, and *R_G_* is an adjustable resistor used to control the gain. The quality factor *Q* can be expressed as shown in Equation (7) and can be adjusted by tuning *R_Q_* and *R_G_*.

(7) $$Q=\frac{1+\frac{R_{4}\left(R_{G}+R_{Q}\right)}{R_{G}R_{Q}}}{1+\frac{R_{2}}{R_{1}}}{\left(\frac{R_{2}R_{F1}C_{1}}{R_{1}R_{F2}C_{2}}\right)}^{1/2}$$

Since the internal gain of the UAF42 is limited to a maximum of 100, the induced signal reaches an amplitude of approximately 0.5 Vpp after band-pass filtering. To further meet the input requirements of the RDC chip, a second-stage inverting operational amplifier is implemented using the remaining internal op-amp of the UAF42, providing additional amplification to achieve the desired signal amplitude.

#### 3.1.3. Resolver to Digital Conversion Circuit

**Figure 8:**
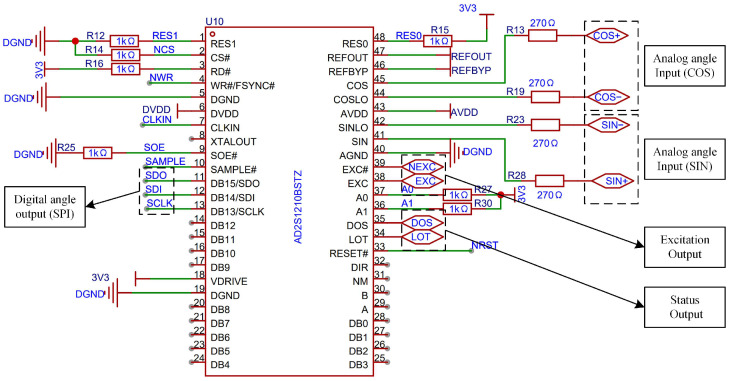
Schematic of the Parallel AD2S1210 Circuit.

In this design, as shown in Figure 8, the selected RDC chip is the AD2S1210 from Texas Instruments (Analog Devices, Norwood, MA, USA). Two chips share a common crystal oscillator, and equal-length, equal-width PCB routing is employed to ensure clock synchronization and minimize phase deviation in the induced signals [26]. The oscillator operates at a frequency of 8.192 MHz, corresponding to an ideal excitation frequency of 10 kHz. The DOS and LOT pins are used for fault detection: when the sine and cosine signals exhibit amplitude mismatch or exceed a defined threshold, the DOS pin outputs a low-level signal; when the internal error of the AD2S1210 surpasses a specified angular threshold, resulting in loss of tracking, the LOT pin outputs a low-level signal. Accordingly, both DOS and LOT pins are connected to external LED indicators to visually indicate system faults. SPI communication is adopted between the AD2S1210 and the control module. As a result, the parallel data pins DB0–DB15 are left unconnected, while the multiplexed pins associated with SPI communication are connected to the microprocessor of the control module for configuration and data exchange.

### 3.2. Software Implementation

#### 3.2.1. RDC Driver Development

**Figure 9:**
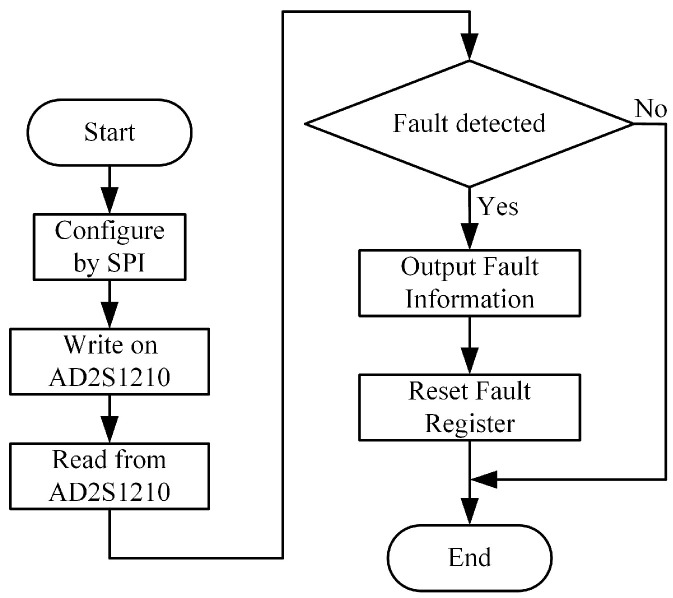
Flowchart of the AD2S1210 Read/Write Program.

The AD2S1210 is capable of decoding the round inductosyn, converting the motor’s angular position and speed information into digital quantities stored in dedicated registers. A software program must be developed to establish communication with the AD2S1210, and the overall process is illustrated in Figure 9.

**Figure 10:**
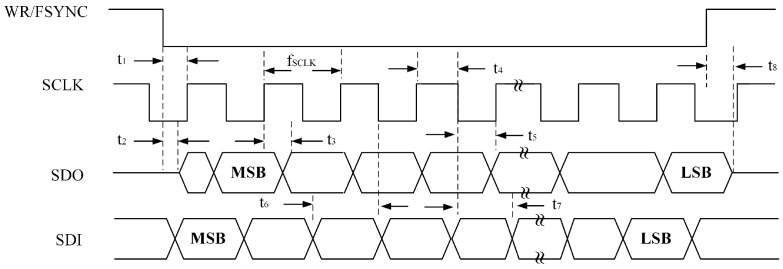
AD2S1210 SPI Read/Write Timing Diagram.

The serial interface of the AD2S1210 consists of four signals: SDO, SDI, WR/FSYNC, and SCLK. The SDI line transfers data to the internal registers, while the SDO line outputs data from them. SCLK serves as the serial clock input, and WR/FSYNC provides frame synchronization. The serial interface does not require a chip-select CS signal; it remains at a low logic level during operation. However, to separately read the coarse and fine channel data of the round inductosyn, the CS pin is used to control whether the chip outputs data to the main controller. The simulated SPI serial communication timing is shown in Figure 10.

**Figure 11:**
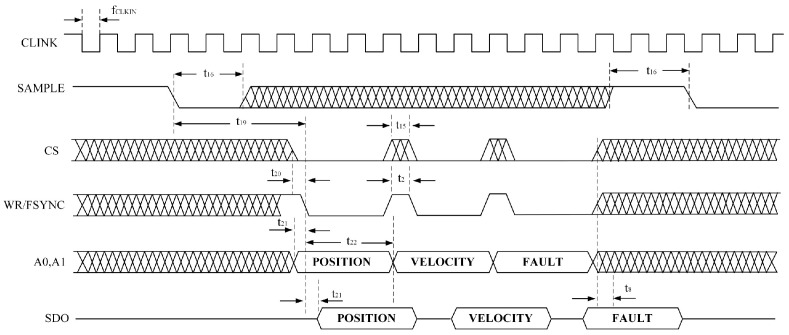
SPI Register Write Timing Diagram.

When the AD2S1210 operates in the normal mode, data transfer is controlled by the serial clock input SCLK, with data shifted out on the rising edge of SCLK. In configuration mode, addressing of internal registers is performed via the SDI input, and data are shifted into the chip on the falling edge of SCLK. The timing diagram for writing configuration registers is illustrated in Figure 11.

**Figure 12:**
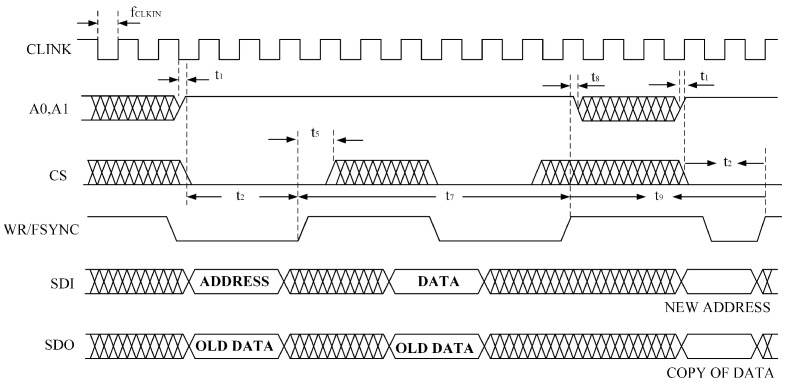
SPI Register Read Timing Diagram.

To read the angular position or rotational speed from the AD2S1210, the SAMPLE signal must first be triggered to update the position and velocity registers. When the SAMPLE signal changes its logic state, data from the AD2S1210’s position and velocity integrators are transferred to their respective registers, while the fault register is also updated simultaneously. By adjusting the logic states of pins A0 and A1, users can select whether the position or velocity data are written to the register and output. The corresponding data timing diagram is shown in Figure 12.

#### 3.2.2. Coarse–Fine Data Fusion

For the dual-channel round inductosyn, the coarse and fine channels have different measurement ranges and resolutions. When readings from both channels are obtained, a data fusion algorithm must be implemented to combine the two. The coarse channel provides the primary positional reference, while the fine channel refines the angular measurement with higher precision. This approach allows the system to achieve both a wide measurement range and high angular resolution, fully utilizing the characteristics of the dual-channel round inductosyn. In this study, the coarse channel has a pole-pair number of N_c_ = 1, and the fine channel has N_f_ = 180, meaning that the fine channel completes 180 electrical cycles within one mechanical revolution of 360°. By combining the two channels, high-resolution angle measurement can be achieved without ambiguity.

**Figure 13:**
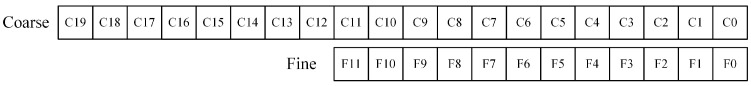
Diagram of Coarse–Fine Channel Alignment and Fusion.

First, the data from both channels are aligned by retaining the 14 most significant bits. The digital angle from the coarse channel is then scaled by a factor of 180 to produce a 28-bit value, ensuring both channels are at the same magnitude level for arithmetic operations. Next, data fusion is performed based on the relationship illustrated in Figure 13. The two most significant bits of the fine channel F11 F10 are compared with bits 11 and 10 of the coarse channel C11 and C10. If F11 = F10 = 00 and C11 = C10 = 11, the upper eight bits of the coarse channel C19~C12 are incremented by one. Conversely, if F11 = F10 = 11 and C11 = C10 = 00, the same bits are decremented by one. Finally, the corrected upper eight bits of the coarse channel C19~C12 are concatenated with the lower 20 bits padded with zeros, and combined with all 12 bits of the fine channel F11~F0 to generate a 22-bit digital value. Dividing this value by the scaling factor of 180 yields the decoded current angular position.

#### 3.2.3. Linear Interpolation and Error Compensation

Although the fusion of coarse and fine channel data enables continuous and unambiguous high-resolution angular output, the round inductosyn still exhibits periodic nonlinear errors due to factors such as winding asymmetry, magnetic path nonuniformity, and amplitude-phase imbalances in the signal amplification and demodulation stages. This error varies periodically within each electrical cycle of the fine channel, with its dominant frequency corresponding to the number of pole pairs N_f_ = 180. To further improve the angular measurement accuracy, a linear-interpolation-based error compensation method is introduced on the basis of the fused angle θ_m_ to eliminate the inherent nonlinearity of the inductosyn.

To accurately establish the angular error model of the round inductosyn, an off-line calibration process is conducted using a high-precision circular optical encoder with a resolution of 720,000 lines as the angular reference. The encoder outputs 720,000 evenly spaced pulses per revolution, corresponding to an angular resolution of approximately 0.0005°, with a measurement accuracy better than ±2″. This ensures sufficient precision for modeling the inductosyn’s angular error.

During calibration, the round inductosyn and the optical encoder are coaxially mounted on a high-precision rotary table, maintaining an alignment error below 10 µm to minimize eccentricity effects. The system drives the rotary table at a constant angular velocity over the 0°~360° range, with the rotational step size Δ*θ_t_* determined by the encoder pulse count. To balance sampling density and data storage requirements, angular data are acquired at 1° intervals, meaning one sampling point of the inductosyn corresponds to every 360 encoder pulses, resulting in N = 2000 total samples.

At each sampling position i, the system simultaneously records two sets of data:

- Reference angle *θ_t_*(*i*): the true mechanical angle calculated from the encoder count;
- Measured angle *θ_m_*(*i*): the angular value obtained from the inductosyn after coarse-fine channel fusion.

The angular error is defined as the difference between the two:

(8) $$e_{i}=\theta_{t}(i)-\theta_{m}(i),\;\;i=0,1,\ldots ,N-1.$$

This produces a discrete error sequence {*e_i_*}. To eliminate the effects of mechanical backlash and rotary table dead zones, a bidirectional measurement approach is employed: the rotary table performs one full revolution in both clockwise and counterclockwise directions, and the mean of the two results is taken as the final error value, expressed as

(9) $$e_{i}=\frac{e_{i}^{\left(\mathrm{cw}\right)}+e_{i}^{\left(\mathrm{ccw}\right)}}{2}$$

## 4. Experimental Verification

### 4.1. Experimental Setup

**Figure 14:**
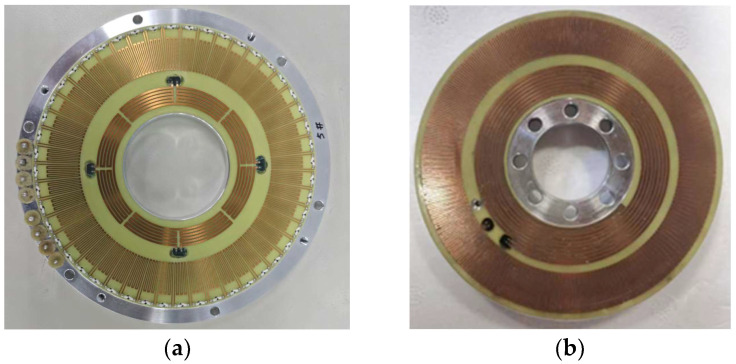
Photograph of the Round Inductosyn: (**a**) Stator of the round inductosyn; (**b**) Rotor of the round inductosyn.

The experimental setup was established to validate the performance of the proposed round inductosyn-based angular measurement system, as shown in Figure 14. The primary objectives were to verify the feasibility of the sine excitation signal, the filtered induced signal, and the RDC conversion circuit. Subsequently, an angular error compensation experiment was performed, in which the round inductosyn and the circular optical encoder were coaxially mounted. The optical encoder was used to measure the angular error of the round inductosyn, and the proposed linear interpolation method was applied for error compensation. The repeatability of the angular measurement was then assessed before and after compensation to determine the effectiveness of the proposed approach.

In this experiment, the round inductosyn used is the JGX 120/360 model developed by Changzhou Weinengda Precision Machinery Co., Ltd. (Changzhou, China),, with a pole-pair ratio of 1:180 between the coarse and fine channels. The reference optical encoder is a Renishaw RSM20USB equipped with a TI20KDA interpolation unit (Renishaw plc, Wotton-under-Edge, Gloucestershire, UK), providing a resolution of up to 720,000 lines per revolution. The driving motor is a J221LWX permanent magnet synchronous motor (Beijing Jingyi Motor Co., Ltd., Beijing, China) with 30 pole pairs and a torque ripple coefficient of less than 3%.

**Figure 15:**
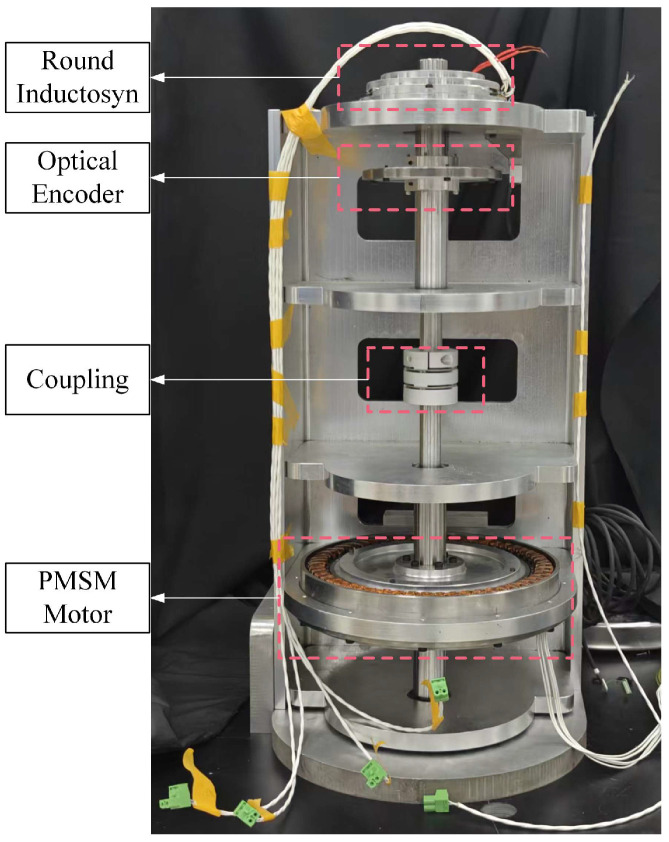
Photograph of the Error Measurement Platform.

As shown in Figure 15, the stator of the round inductosyn is fixed to an aluminum alloy base. The distance between the rotor and stator planes is adjusted to be no less than 0.02 mm, and a dial gauge is used to correct the coaxial alignment between the rotor and the shaft before securing the rotor. Finally, the stator is fastened to the upper cover using bolts to ensure structural stability.

**Figure 16:**
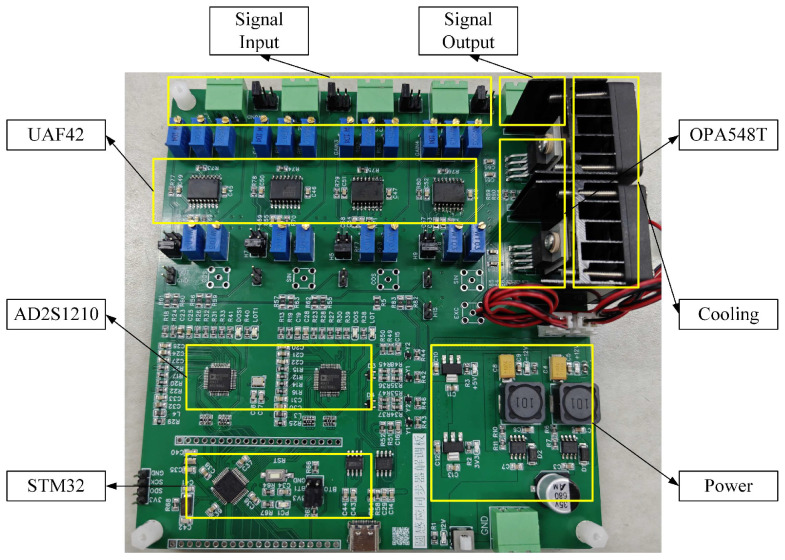
Digital demodulation PCB board.

Figure 16 shows the hardware circuit board of the angular measurement system. The excitation signal is connected to the stator side of the upper cover through a twisted and shielded pair of wires, while the output terminals of the inductosyn are connected to the input ports of the UAF42 filter circuit. The PCB is divided into three functional sections: a digital section, an analog section, and a power supply section. These sections are connected through 0 Ω resistors to achieve single-point grounding.

### 4.2. Hardware Signal Measurement

#### 4.2.1. Excitation Signal Power Amplification Test

**Figure 17:**
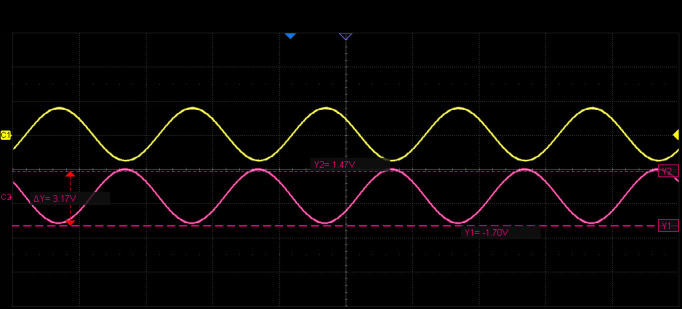
Waveforms of the Excitation Signal Before and After Power Amplification.

The excitation signal is generated by the AD2S1210 and consists of dual differential sinusoidal waves at 10 kHz with a peak amplitude of 5.9 V. Each channel includes a DC offset of 2.5 V as a reference voltage. As shown in Figure 17, Channel 1 represents the original sine wave output from the AD2S1210, with a frequency of 10 kHz and a peak-to-peak amplitude of 6.8 Vpp, while Channel 2 shows the excitation signal after power amplification, maintaining the same 10 kHz frequency with an amplitude of 7.4 Vpp. A 90° phase shift occurs between the pre- and post-amplified signals, with a slight increase in voltage amplitude but no change in frequency, which is consistent with the theoretical design requirements.

#### 4.2.2. Induced Signal Filtering and Amplification

**Figure 18:**
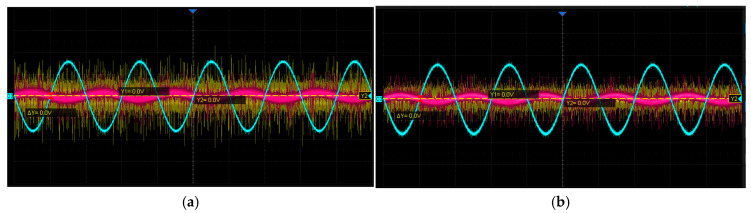
Waveforms of the Induced Signals Before and After Processing: (**a**) Coarse-channel sine signal; (**b**) Coarse-channel cosine signal.

Since the original induced signals generated by the round inductosyn are extremely weak, twisted-pair shielded cables are used for signal transmission to minimize interference during propagation. This configuration effectively exploits the differential nature of the signals and enhances their noise immunity. The induced signal amplification module amplifies the two millivolt-level signals to 3.2 Vpp, ensuring that their phases and peak amplitudes remain nearly identical, with a phase deviation of less than 44° from the excitation signal. The coarse and fine channels operate independently but follow identical processing procedures. The sine and cosine induced signals of the coarse channel were selected and measured using an oscilloscope, both before and after filtering and amplification, as shown in Figure 18.

Figure 18a,b show the sine and cosine signals of the coarse channel, respectively. In each figure, CH1 represents the original induced signal, CH2 denotes the signal after the first-stage amplification, and CH3 corresponds to the signal after band-pass filtering and second-stage amplification. As shown in Figure 18a, the amplitude of the original signal is approximately 10 mV; after the first amplification stage, it increases to about 100 mV, and following band-pass filtering and second-stage amplification, the amplitude reaches 3.16 Vpp with reduced noise. Similarly, in Figure 18b, the original signal amplitude is about 5 mV, rising to approximately 100 mV after the first stage and to 3.08 Vpp after filtering and the second stage, also with noticeable noise suppression. After undergoing first-stage amplification, band-pass filtering, and second-stage amplification, the processed signals exhibit amplitudes within the range of 3.2 ± 0.6 Vpp, a frequency of approximately 10 kHz, and smooth, well-defined sine and cosine waveforms that meet the design specifications.

**Figure 19:**
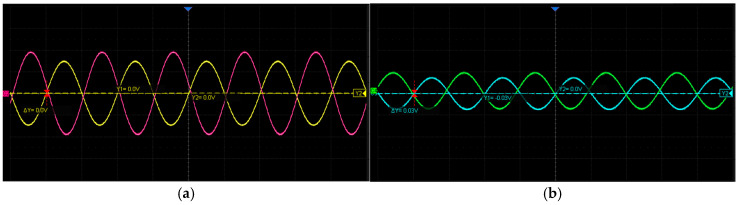
Filtered and Amplified Sine and Cosine Waveforms of the Coarse and Fine Channels: (**a**) Coarse-channel sine and cosine induced signals; (**b**) Fine-channel sine and cosine induced signals.

As shown in Figure 19, the sine and cosine signals of both the coarse and fine channels are mutually orthogonal, each with a frequency of approximately 10 kHz. Within the allowable error range, the maximum and minimum amplitudes are equal, and the signal amplitudes vary sinusoidally with the rotational angle, consistent with the theoretical predictions.

### 4.3. Experimental Validation and Statistical Analysis

**Figure 20:**
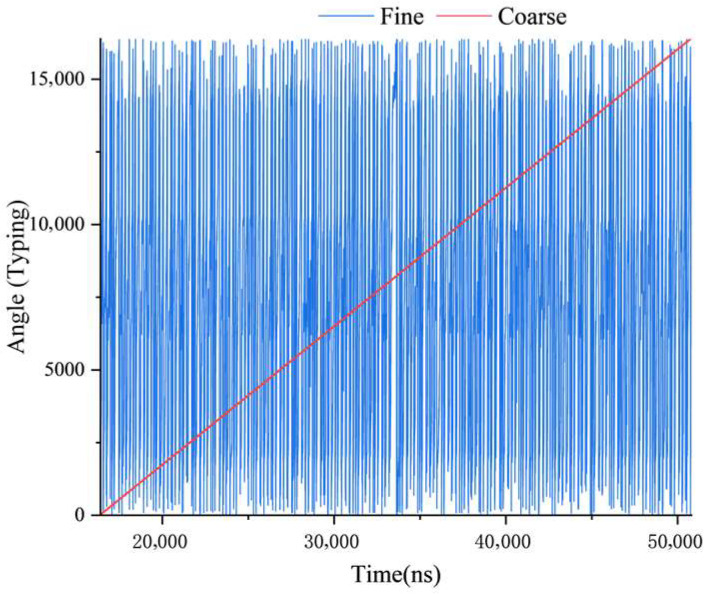
Position Feedback Values of the Coarse and Fine Channels.

During the drive test, the permanent-magnet cylindrical synchronous motor rotated at a constant speed while the angular position was measured. In this design, both the coarse and fine channels were configured with 14-bit resolution, corresponding to position values ranging from 0 to 16,384. The feedback of the position data, shown in Figure 20, indicates that the data transmission path functions correctly.

The proposed round inductosyn–based angular measurement system was experimentally validated using a high-precision Renishaw circular optical encoder as the reference. To comprehensively evaluate measurement accuracy and repeatability, six independent calibration experiments were conducted under identical experimental conditions.

In each experiment, the angular output of the round inductosyn was compared with the reference encoder over a full mechanical revolution. One sampling trigger was generated for every 360 pulses of the optical encoder, resulting in 2000 uniformly distributed sampling points over a 360° rotation. The previously described coarse–fine fusion algorithm was applied to generate absolute angular output with a 20-bit resolution, where the upper 8 bits represent the coarse-channel cycle number and the lower 14 bits correspond to the fine-channel position within each cycle.

**Figure 21:**
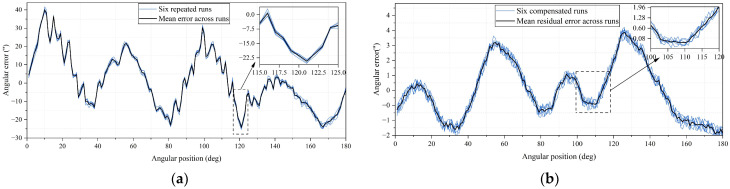
Angular error from six repeated experiments before and after compensation: (**a**) Before compensation; (**b**) After compensation.

The angular error distributions of the six repeated experiments before and after linear interpolation compensation are superimposed in Figure 21a,b, respectively. Before compensation, all six error curves exhibit highly consistent shapes but relatively large amplitudes, indicating the presence of stable and repeatable systematic errors. After applying linear interpolation compensation, the error magnitude is significantly reduced, and the six curves closely overlap, demonstrating both improved accuracy and good inter-run consistency.

To quantitatively assess the experimental results, statistical metrics including mean error, root mean square (RMS) error, standard deviation (STD), and maximum absolute error were calculated for each experimental run. The summarized results are presented in Table 1. Before compensation, the RMS error across the six runs is approximately 15″, with maximum absolute errors exceeding 40″. After compensation, the RMS error is reduced to approximately 1.52″, and the maximum residual error is constrained within ±3.8″.

In addition, repeatability across the six experiments was evaluated by computing the standard deviation of angular error across runs at each sampling position. The statistical results are summarized in Table 2, where the mean cross-run standard deviation is reduced from 0.94″ before compensation to 0.23″ after compensation, and the 95th percentile value decreases from 1.40″ to 0.29″.

Overall, the experimental results confirm that the proposed round inductosyn-based angular measurement system operates reliably and achieves high angular accuracy through the combined effect of hardware design and software algorithms. The excitation, signal conditioning, and resolver-to-digital conversion circuits function as expected, and the coarse–fine fusion together with linear interpolation compensation effectively suppress systematic errors. The system achieves sub-4″ peak error with excellent continuity and repeatability, demonstrating its suitability for high-precision servo control and aerospace attitude measurement applications.

## 5. Conclusions

This study presented the design, implementation, and experimental validation of a high-precision angular measurement system based on a round inductosyn. The proposed system integrates both hardware and software innovations to achieve accurate and stable angular detection suitable for precision servo control and aerospace applications. In particular, the system performance was evaluated through repeated experiments to assess both accuracy and repeatability.

The working principle of the round inductosyn was analyzed in detail, and the corresponding hardware circuitry, including the excitation power amplifier, induced signal filter and amplifier, and resolver-to-digital conversion module, was designed and verified. The software framework was developed to drive the RDC chip, perform coarse–fine channel data fusion, and execute linear interpolation-based error compensation. Experimental results demonstrated that the hardware operated reliably and the software algorithms effectively enhanced the measurement precision. Based on six independent experimental runs over a full rotation, the compensated angular error exhibited consistent behavior across trials. After compensation, the residual angular error was within ±3″, and the output exhibited excellent smoothness and stability, consistent with theoretical expectations.

The proposed method successfully combines wide-range measurement capability with high angular resolution by leveraging the dual-channel structure of the round inductosyn. The implementation verified that the system effectively suppresses non-linear errors caused by amplitude and phase imbalance. Quantitative repeatability analysis further showed that the mean cross-run standard deviation was reduced from sub-arcsecond levels before compensation to approximately 0.23″ after compensation, with a corresponding reduction in dispersion across the full rotation, demonstrating improved robustness and repeatability.

In summary, the developed round inductosyn-based angular measurement system demonstrates high accuracy, stability, and practical feasibility. The consistency observed across repeated experiments confirms that the proposed compensation strategy enhances not only accuracy but also measurement repeatability, offering a promising approach for high-precision servo mechanisms and attitude measurement in aerospace applications.

## Figures and Tables

**Table 1:** Statistical error metrics of six repeated experiments before and after compensation.

Run		Mean (″)	RMS (″)	STD (″)	MAX|err|(″)
Before Compensation	Run1	0.839433878	15.19007234	15.20916673	39.864069
	Run2	1.019976133	15.13702277	15.14474654	39.870046
	Run3	0.965201183	15.28328626	15.29532382	39.365431
	Run4	1.089516306	15.13408292	15.1369199	40.6284
	Run5	0.954129094	15.03400833	15.04555242	38.961935
	Run6	1.105046756	15.14263725	15.14438891	41.710066
After Compensation	Run1	0.355368044	1.522271424	1.484339651	3.62336
	Run2	0.3846828	1.517288345	1.471807599	3.682352
	Run3	0.3758228	1.521985025	1.478968414	3.767832
	Run4	0.395965378	1.521528844	1.47320018	3.644992
	Run5	0.374393556	1.51674012	1.473906015	3.547176
	Run6	0.398513289	1.506642151	1.457035314	3.649072

**Table 2:** Cross-Run Repeatability Statistics of Angular Error Before and After Compensation.

Condition	Mean Cross-Run STD (″)	95th Percentile STD (″)	Max STD (″)
Before compensation	0.94	1.40	1.85
After compensation	0.23	0.29	0.34

## Data Availability

All the relevant data supporting the findings of this study are available within the paper.
